# Pathogenicity and virulence of *Giardia intestinalis*

**DOI:** 10.1080/21505594.2026.2745308

**Published:** 2026-10-06

**Authors:** Anjeela Bhetwal, Emma Stenlund, Staffan G. Svärd

**Affiliations:** Department of Cell and Molecular Biology, Uppsala University, Uppsala, Sweden

**Keywords:** Diarrhea, protozoa, vesicle, protease, microbiome

## Abstract

Giardiasis, caused by *Giardia intestinalis*, is a prevalent intestinal protozoan infection worldwide. Clinical manifestations are variable, and many infections remain asymptomatic. Disease pathogenesis is multifactorial, involving brush border enzyme deficiency, apoptosis, dysbiosis and increased intestinal permeability. Advances in genomics, genetic tools, and model systems have substantially improved our understanding of the parasite’s virulence mechanisms. *Giardia* exhibits considerable genetic diversity, with distinct assemblages associated with differences in parasite biology. Attachment is mediated by the ventral disk, while the parasite’s eight flagella contribute to motility and intestinal persistence. Antigenic variation of cysteine-rich surface proteins enables immune evasion and promotes chronic infection, but the level of inflammation is low. The parasite releases a variety of proteins and extracellular vesicles containing proteins and small RNAs that play important roles in host-cell interactions and modulation of the gut microbiome. This review describes areas that need further research to connect pathogenesis to specific virulence mechanisms.

## Introduction

*Giardia intestinalis* (also called *Giardia lamblia* or *Giardia duodenalis*) is one of the most globally widespread intestinal protozoan parasites of humans. Antonie van Leeuwenhoek first observed it in 1681 in his own stool, making it likely the first human intestinal parasite examined microscopically [1]. *Giardia* is a major contributor to the global burden of water‑borne gastrointestinal disease. In 2021 the mean number of symptomatic *Giardia* cases (giardiasis) was estimated at 321 million cases worldwide, with the highest incidence among young children and vulnerable populations in regions with limited access to safe drinking water and adequate sanitation [2]. The prevalence of giardiasis varies widely by region, reaching 10–40% in low- and middle-income countries, while remaining lower but persistent (1–5%) in high-income countries, where symptomatic cases are primarily linked to travel and waterborne outbreaks [3,4]. It should be noted here that most *Giardia* infections are asymptomatic and improved diagnostic methods using molecular analyses [5] and metagenomics [6] of stool samples have shown higher prevalences also in high income countries. Still, even with underestimations of the number of *Giardia*-infected people in the European Union, giardiasis remains an important public health concern, with 10,894 cases reported in 2022 and a significant disease burden of around 1.2 million disability-adjusted life years annually, highlighting its continued role in both sporadic and outbreak-related diarrheal diseases [7].

Infections with *Giardia* are typically acquired through ingestion of infective cyst-forms (Figure 1) in contaminated drinking water or food or via direct fecal-oral transmission. Giardiasis is clinically characterized by a broad range of outcomes, from asymptomatic carriage to symptomatic disease. When present, symptoms commonly include watery diarrhea, abdominal pain and cramping, bloating, nausea, malabsorption, and weight loss [8]. This variability reflects multiple contributing factors, including host immune status, age, nutritional condition, parasite genotype, infectious dose, and the presence of co‑infections [9]. As *Giardia* infection disrupts intestinal physiology, clinical symptoms are due to nutrient malabsorption, impaired digestion, disrupted brush border enzymes, shortening of brush border microvilli, and electrolyte imbalance [8,10]. Infections can also develop into chronic functional gastrointestinal disorders, such as post-infectious irritable bowel syndrome (PI-IBS) [11], and the parasite can alter the gut microbiome during both acute and persistent infections [12]. Moreover, chronic giardiasis can contribute to stunted growth, reduced weight‑for‑age, anemia, and impaired cognitive development, particularly in young children in low‑resource settings [13]. Metronidazole (Flagyl) is the current first-line therapy of giardiasis, but treatment failures are increasing world-wide and there are few alternative treatments [14]. Thus, there is a need for further understanding of resistance mechanisms and development of alternative treatments.

**Figure 1.**
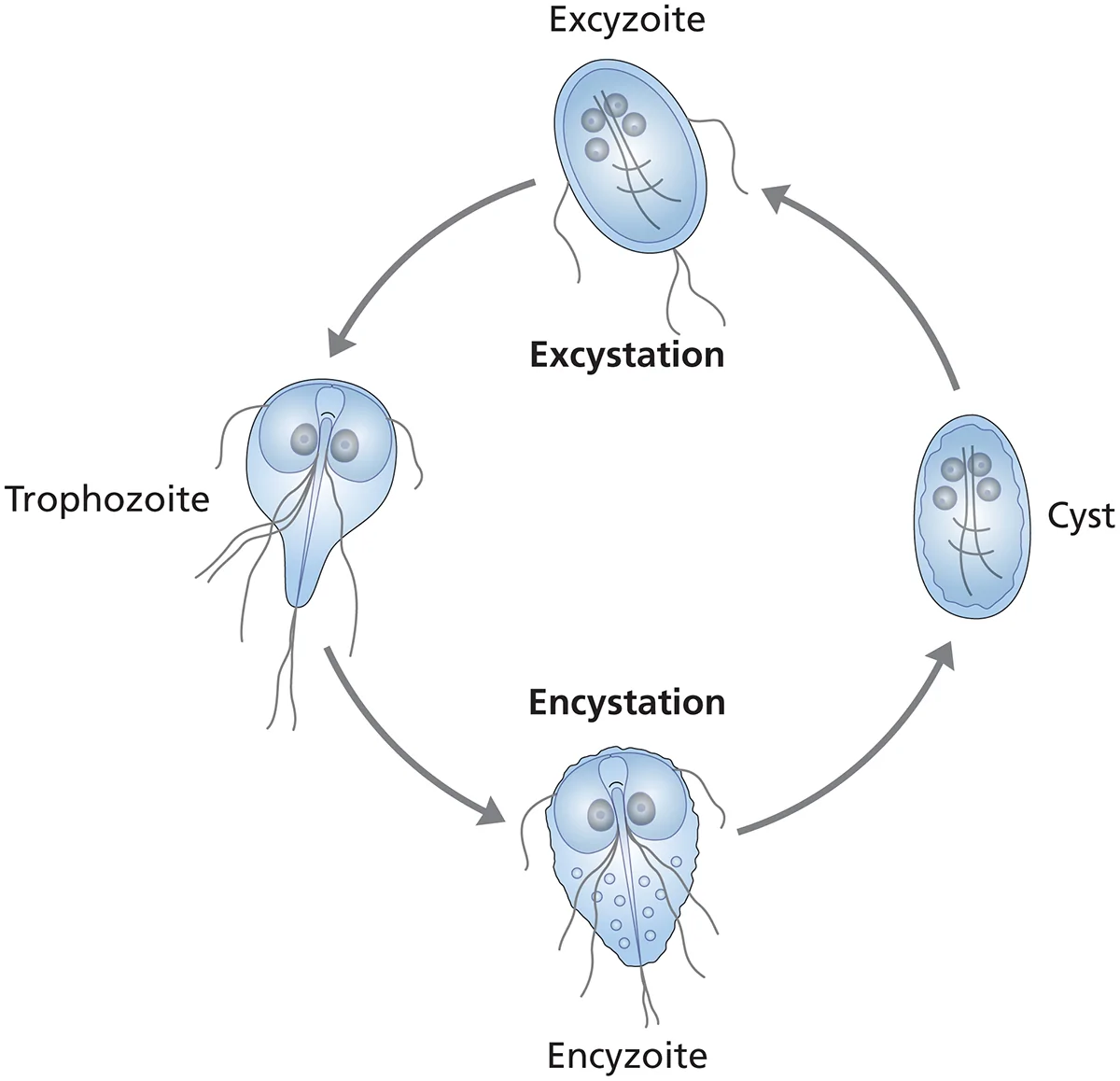
The *Giardia* life-cycle. *Giardia* has a simple life-cycle with infectious cysts and disease-causing trophozoites. The ploidy changes during the differentiation process. Cysts, with four tetraploid nuclei, are formed from trophozoites in the encystation process with encyzoites (two tetraploid nuclei) as intermediary, transient forms. *In vitro* encystation is induced by high bile and slightly alkaline pH. Cysts can be excysted by an initial acid treatment followed by addition of trypsin. This releases the transient excyzoites (four tetraploid nuclei) that quickly divide twice to generate four trophozoites with two tetraploid nuclei.

Although *Giardia* has long been recognized as a human pathogen, the molecular mechanisms that enable it to establish infection and cause disease have only begun to be clarified in the recent decades. The genome sequencing of *Giardia* revealed that the parasite encodes a small, yet highly effective repertoire of factors that can destabilize the intestinal micro-environment [15,16]. Subsequent molecular investigations using comparative genomics, transcriptomics, proteomics and precise-functional analyses of virulence-related molecules have further broadened our understanding how *Giardia* regulates the secretion of effector molecules, modulates epithelial barrier function, regulates antigenic variation, alters host signaling and adapts to the intestinal niche [1,17].

This review summarizes recent advances in *Giardia* pathogenesis and virulence, beginning with the parasite’s life cycle and the mechanisms of intestinal infection, including mucosal damage, immune evasion, and the deployment of key virulence determinants. We try to identify limitations in current knowledge and identify new research questions that need more focus in the future.

## Biology, life cycle and intestinal colonization

*Giardia intestinalis* is a flagellated, anaerobic protozoan parasite and represents the only known diplomonad that infects humans. It lacks true mitochondria, Golgi-apparatus and peroxisomes but contains reduced mitochondrial remnants called mitosomes, which play a role in iron-sulfur cluster assembly but lack respiratory or ATP-generating functions [18]. Genomic analyses show that *Giardia* retains mitochondria-related genes, indicating that it has undergone secondary genome reduction rather than representing a primitively simple eukaryote [16,19]. At the genetic and metabolic level, *Giardia* displays extreme reductive evolution; its compact ~12 Mb genome encodes a remarkably minimalistic proteome, lacking or having heavily reduced pathways for biosynthesis of vitamins, nucleotides, amino acids, and complex lipids, which forces complete dependence on host-derived nutrients from the intestinal lumen [1,16]. Notably, it lacks a complete tricarboxylic acid cycle and oxidative phosphorylation machinery, relying instead on glycolysis and arginine catabolism via the arginine dihydrolase pathway for ATP generation [1,16]. *Giardia* converts host L-arginine to citrulline and ornithine while producing ATP and depleting L-arginine needed for host nitric oxide synthesis, thereby suppressing mucosal immunity and illustrating the parasite’s efficient integration of metabolism and immune evasion [20,21]. The competition for different types of nutrients with the host cells in the small intestine results in reduced nutrient uptake in the host since the parasite’s enzymes and transporters are more efficient [1,22]. This, in turn, results in metabolic remodeling in the host, effects on the bacterial microbiome and reduced growth of the infected host [13]. The effects of *Giardia*’s metabolism on the host’s and its intestinal microbiome are an area that needs more focus if we are to understand *Giardia*’s pathogenic effects.

Molecular studies have defined the taxonomy of *G. intestinalis*, identifying eight distinct genetic assemblages (A-H) through multilocus genotyping and genome sequencing [23]. Of these, assemblages A and B are routinely found in humans and occasionally in other mammals. The sequence identity between assemblage A and B is only around 75% at the nucleotide level, suggesting that they are two different species [24–26]. Isolates from assemblage A are further divided into three major groups (A-I, A-II and A-III) based on DNA sequence similarities, with sub-assemblage A-I exhibiting a broad zoonotic potential and A-II being predominantly associated with human colonization and infections [24]. Recent data suggest that A-I isolates are clonal and show little genetic variation world-wide, whereas A-II isolates are non-clonal and much more variable, even showing signs of sexual recombination [24]. In contrast, assemblages C through H demonstrate pronounced host-specificity, being largely restricted to dogs (C/D), cats (F), livestock (E), and marine mammals (G/H), reflecting long-term co-evolution with their vertebrate hosts [1,27]. Moreover, some assemblages can also be transmitted to humans through contact with the feces of infected animals or contaminated water, leading to an overlap between human and animal strains and enabling the same parasite species to infect both humans and animals [3]. Different *Giardia* genotypes/assemblages have been suggested to have different effects on the host [27]. This is reasonable, considering the genetic differences and host-specificities, but more conclusive data is needed to show this and what it means for the pathogenicity [27]. It will require stronger molecular epidemiological data connected to experiments performed with *Giardia* isolates from different assemblages and the use of different model systems *in vitro* and *in vivo*.

The life cycle of *Giardia* alternates between two morphologically and functionally distinct stages: i) the metabolically active trophozoite (Figure 1) and ii) the environmentally robust cyst (Figure 1). The trophozoite of *G. intestinalis* is the actively dividing, flagellated form that colonizes the proximal small intestine, particularly the duodenum and upper jejunum [10]. The trophozoite is a motile, pear-shaped protozoan measuring about 12–15 μm long and 6–8 μm wide, featuring four pairs of flagella (anterior, posterolateral, caudal, and ventral) and a ventral disk (Figure 1) that facilitate attachment to the intestinal epithelium [28]. It has two transcriptionally active nuclei surrounded by ER and ribosomes, as well as a the “smile-like” median body of uncertain function. The cyst measures 7–10 μm in length and 5 μm in width (Figure 1), with a chemically resistant, bilayered wall composed of β-1,3-N-acetylgalactosamine polymers (giardan) and three major cyst wall proteins (CWP-1 to-3) [29]. The mature cysts contain four nuclei instead of the two nuclei present in trophozoites (Figure 1). Its low metabolic activity (only ~10–20% of trophozoite) enables prolonged survival in cool, moist environments, facilitating transmission and persistence in nature [1,30].

Infection of the susceptible host is initiated upon ingestion of as few as 10–100 mature, quadrinucleate cysts [29]. Upon reaching the stomach, gastric acid destabilizes the cyst wall and primes the parasite for excystation (Figure 1). Subsequent exposure to pancreatic proteases and bile salts in the proximal duodenum triggers cytoskeletal reorganization and flagellar activation. Each excysting cyst releases one tetranucleated excyzoite (Figure 1), which rapidly divides twice, generating four replication-competent trophozoites ready to establish intestinal colonization [1,29]. The excystation process of *Giardia* is understudied due to experimental constraints but early transcriptomic analyses identified 71 genes that are specifically up-regulated during the process [31]. Further studies using modern genetic tools to generate mutants in key genes and transcriptomic analyses at the single cell level are needed to define the different stages of excystation.

Trophozoites generated from excystation then inhabit the proximal small intestine without invading the intestinal lining. They reside on the epithelial surface, where they consume nutrients from the intestinal fluid and replicate asexually by binary fission. This process rapidly expands the parasite population within the host while exploiting abundant bile salts, dietary lipids and other nutrients through salvage pathways [32]. Experimental infections in mice show that *Giardia* colonizes the small intestine in discrete foci with higher densities in the upper small intestine and no colonization in the large intestine [33]. Early data suggested that encystation was induced in the distal end of the small intestine, due to starvation of nutrients like cholesterol and other lipids [34,35]. However, data generated in mice and gerbils using luciferase-labeled parasites (both assemblage A and B) suggest that parasite-density is important [33,36]. Encystation is patchy in the different foci and can start already in the upper part of the small intestine but with a higher density in mid-small intestine [33,36]. Several stimuli seem to be important for induction of encystation: pH (slightly alkaline [35]), lipid concentrations (high primary bile salts [35]-low cholesterol [34]), glucose (high [37]) and arginine levels (high [20]). Encystation *in vitro* involves exposing parasites to high levels of bile in a slightly alkaline environment [35] but not all *Giardia* isolates encyst under these conditions, and the excystation levels of the generated cysts are quite low, suggesting that there are signals in the *in vitro* encystation conditions that are missing. Clearly, the encystation stimuli and their interactions need to be further investigated to fully understand how *Giardia* encystation is induced. It is also possible that encysting parasites in high-density foci higher up in the small intestine have a role in disease-induction. The use of luciferase‑labeled parasites with mutations in key genes for encystation can reveal how the process is regulated *in vivo* and can also show whether it affects *Giardia*’s pathogenicity.

The encystation stimuli induce membrane reorganization, trigger adenylate cyclase 2-dependent cAMP upregulation [38], and initiate encystation-specific transcription of the CWP-1 to 3 genes and enzymes producing giardan [39,40]. Recent data show that these encystation-specific genes are regulated by both transcriptional activators (MYB2) [41] and repressors (GLP4) [42]. The cyst-wall proteins are transported from ER exit sites [43] to the encysting parasite surface (enzyzoite, Figure 1) via encystation-specific vesicles (ESVs) and there they bind the giardan fibers via lectin activities [44]. The resulting cysts are environmentally persistent and optimized for fecal-oral transmission [32]. The resilience of *Giardia* cysts makes giardiasis extremely difficult to control because the cysts can survive for months in cold water environments (Table 1). They also resist standard chlorination of water, allowing them to persist and transmit infection long after shedding from the host. A low infectious dose (as few as 10 cysts), combined with this environmental stability, makes *Giardia* highly transmissible via contaminated drinking water, food, fomites, and direct person-to-person or zoonotic contact [3,29]. Recognizing its importance, interfering with the encystation process could interrupt transmission, and several encystation-associated proteins are being investigated as potential drug and/or vaccine targets [45].

**Table 1.** Summary of representative virulence factors of *Giardia intestinalis* and their biological roles during pathogenesis.

Virulence factors	Representative members	Biological role	Mechanism of pathogenesis	References
Cyst, Cyst formation (encystation)	Cyst wall proteins 1–3, Enzymes producing giardan, MYB2 and GLP4 transcription factors	Transmission, survival outside of the host	Production of resistant cysts that can be transmitted to new hosts; low infectious dose (10–100 cysts).	[31–35,37–42,44]
Ventral disk & disk-associated proteins (DAPs)	Tubulin core; >90 DAPs (e.g. Median Body Protein, MBP)	Primary attachment organelle; mediates reversible colonization of the epithelium	Disk contraction/suction (hydrodynamic model) forms a seal with enterocytes; CRISPR knockout of MBP flattens the disk, impairs shear resistance and reduces epithelial barrier disruption.	[46–54]
Flagella	Axonemal/flagellar proteins	Motility; proposed accessory role in disc attachment	Ventral flagellar beating suggested to generate negative pressure under the disk (hydrodynamic model); attraction of nutrients; penetration of mucus; inhibition of flagellar motility reduces mucosal damage.	[47,51,52,57,172]
Giardins (cytoskeletal family)	α-, β-, γ-, δ-giardins	Structural stabilization of the ventral disk; contributes to motility, cytokinesis and adhesion	Stabilize the microtubule/microribbon/crossbridge array of the disk; several are secreted and immunoreactive in patient sera; surface localized.	[45,47,61,169,172–174,176,185]
Alpha-1 giardin (taglin)	α-giardin family (annexin-like)	Lectin-like, disk-independent adhesion, especially in excyzoites lacking a mature disk	Ca^2+^-dependent binding to acidic phospholipids, mucins and enterocyte glycans; secreted via an unconventional (UPS) pathway despite lacking a signal peptide.	[60,61,144,169,174,175]
Extracellular vesicles (EVs)	Small vesicles (<100 nm) & large vesicles (100–400 nm)	Delivery vehicle for multiple virulence factors and small RNAs; host- and parasite-directed communication	Carry α-giardins, VSPs, CRMPs and metabolic enzymes to host cells; deliver small RNAs that alter gene expression in bacteria, other parasites and host cells; large-EV formation requires peptidyl-arginine deiminase (PAD) activity.	[141,145–151]
Variant-specific surface proteins (VSPs)	~136 members (e.g. VSP9B10A, VSPH7, VSPAS7)	Immune evasion/ antigenic variation; some members also act as adhesins or cytotoxic proteases	Only one VSP surface-expressed at a time via RNAi-based switching (ARCS: antibody-induced clustering → microvesicle shedding → calcium-dependent switch); protects against luminal proteases; VSP9B10A shows cathepsin-B-like proteolytic/cytotoxic activity; VSPAS7 dampens macrophage NF-κB/ERK signaling.	[115,118–128,131]
Cysteine-rich membrane proteins (CRMPs/HCMPs)	≥94 members; ~12 secreted	Surface diversification and strain/host adaptation	Multiple CRMPs co-expressed simultaneously (unlike VSPs); expression modulated by iron availability and histone acetylation; genotype- and strain-specific secretion patterns.	[45,115,116,137,138,161]
Secretory cysteine-rich proteins (SCRPs, tenascin-like)	~20 genes; up to 7 detected in the secretome	Proposed disruption of mucosal repair via EGF-receptor competition	Following mucus degradation by cathepsin B and a secreted nuclease, SCRPs are proposed to competitively ligate exposed EGF receptors via EGF-like domains, promoting apoptosis and barrier disruption.	[115,138,140]
Secreted cysteine proteases (cathepsins)	Giardipain-1/CP14019, CP16160, CP16779; ~25 members of CP family	Epithelial injury, barrier disruption, immune evasion	Cleave tight/adherens-junction proteins (claudins, occludin, JAM-1, β-catenin, E-cadherin) and villin; degrade chemokines (CXCL1-3, CXCL8, CCL2/20), sIgA and β-defensin 1; alter mucin glycosylation; induce enterocyte apoptosis; degrade bacterial biofilm matrix.	[67,68,136–138,152–157]
Arginine deiminase (gADI)	GL50803_112103	Metabolic competition and immunosuppression (“warginine” strategy)	Depletes extracellular L-arginine (substrate of host NO synthase); suppresses nitric-oxide production and neutrophil killing; skews dendritic cells (↑TNF-α, ↓IL-10/IL-12p40); suppresses T-cell proliferation; activates mast cells.	[20,21,137,138,162,165–167,169,170]
Ornithinecarbamoyltransferase (gOCT)	GL50803_10311	Supports arginine dihydrolase pathway (ATP generation); strong immunogen	Generates L-ornithine that fuels the arginine/ornithine antiporter sustaining gADI-driven arginine depletion.	[137,162,169–171]
Enolase (gENO)	GL50803_11118	Moonlighting glycolytic enzyme; host tissue remodeling	Binds and activates host plasminogen to plasmin, promoting extracellular-matrix degradation; recombinant gENO induces cytopathic effects in enterocytes.	[137,162,163,169,170]
Toxin-like molecules	CRP136/VSP188; gESP58	Enterotoxin-like activity	CRP136/VSP188 contains sarafotoxin-like repeats predicted to activate endothelin receptor A, enhancing smooth-muscle contraction; gESP58 increases epithelial permeability and induces fluid secretion via a proposed cholera-toxin-like (GM_1_-binding, cAMP/PKA, Cl^−^-efflux) pathway.	[9,177–180]

Abbreviations: ADHP, arginine dihydrolase pathway; CP, cysteine protease; CRMP/HCMP, cysteine-rich/high-cysteine membrane protein; DAP, disk-associated protein; ES, excretory-secretory; EV, extracellular vesicle; MBP, Median Body Protein; NO, nitric oxide; PAD, peptidyl-arginine deiminase; SCRP, secretory cysteine-rich protein; VSP, variant-specific surface protein.

## Host-parasite interplay in pathogenesis of *giardia*

### Adhesion to the intestinal epithelium

Adhesion of trophozoites to the intestinal epithelium is a critical early step in *Giardia* pathogenesis (Figure 2), allowing the parasite to establish colonization despite strong peristaltic forces and rapid turnover of the enterocyte layer. Trophozoites attach in a fully reversible manner, allowing repeated cycles of detachment and re-attachment that facilitate their spread across the intestinal mucosa. This attachment is mediated primarily by the ventral disk, a concave, specialized dome-shaped organelle rich in microtubules that occupies the ventral surface of the trophozoite (Figure 1 and 2). The disk is composed of tubulin and more than 90 disc-associated proteins (DAPs), many of which are unique to *Giardia* [46–50]. A long-standing question in the *Giardia* field has been whether the flagella are needed for attachment with the disk. Early in *Giardia* research a model for attachment (the hydrodynamic model) was proposed by Holberton [51] suggesting that ventral flagellar beating generates a negative pressure under the disk and in that way suction forces. This idea was recently supported by Picou *et al*. using biophysical assays and modeling [52]. However, disk attachment appears to be a more complicated and dynamic process. Disk-mediated attachment likely follows a stepwise process of surface contact, beginning with a loose interaction with the ventrolateral flange [53] and progressively increasing the area of cellular surface contacts as the disk forms a seal and enables resistance to shear stresses [54]. Dynamic disk contraction is essential for maintaining stable attachment and it contributes to epithelial injury (Figure 2 and Table 1), as inhibition of disk or flagellar motility markedly reduces mucosal damage [47,49]. CRISPR/Cas9-based systems have only recently been developed in *Giardia* [55,56], enabling generation of targeted mutants and complete gene knockouts of non-essential genes. Complete CRISPR-mediated knockouts of the abundant disc-associated protein MBP revealed flattened disks, abnormal surface interactions, and an inability to withstand shear forces under fluid flow; the parasite also showed reduced ability to disrupt the epithelial barrier [49]. High definition DIC imaging of *Giardia* trophozoites on human adult stem-cell‑derived epithelial cell layers show that *Giardia* can be loosely attached via the disk while simultaneously moving over the intestinal epithelial cell (IEC) surface [57]. Further studies of knockout mutants in disk and flagellar proteins have the potential to reveal additional details of disk-mediated attachment and its connection to flagellar activity, but even now we can see that it is a multifactorial and unique attachment process.

**Figure 2.**
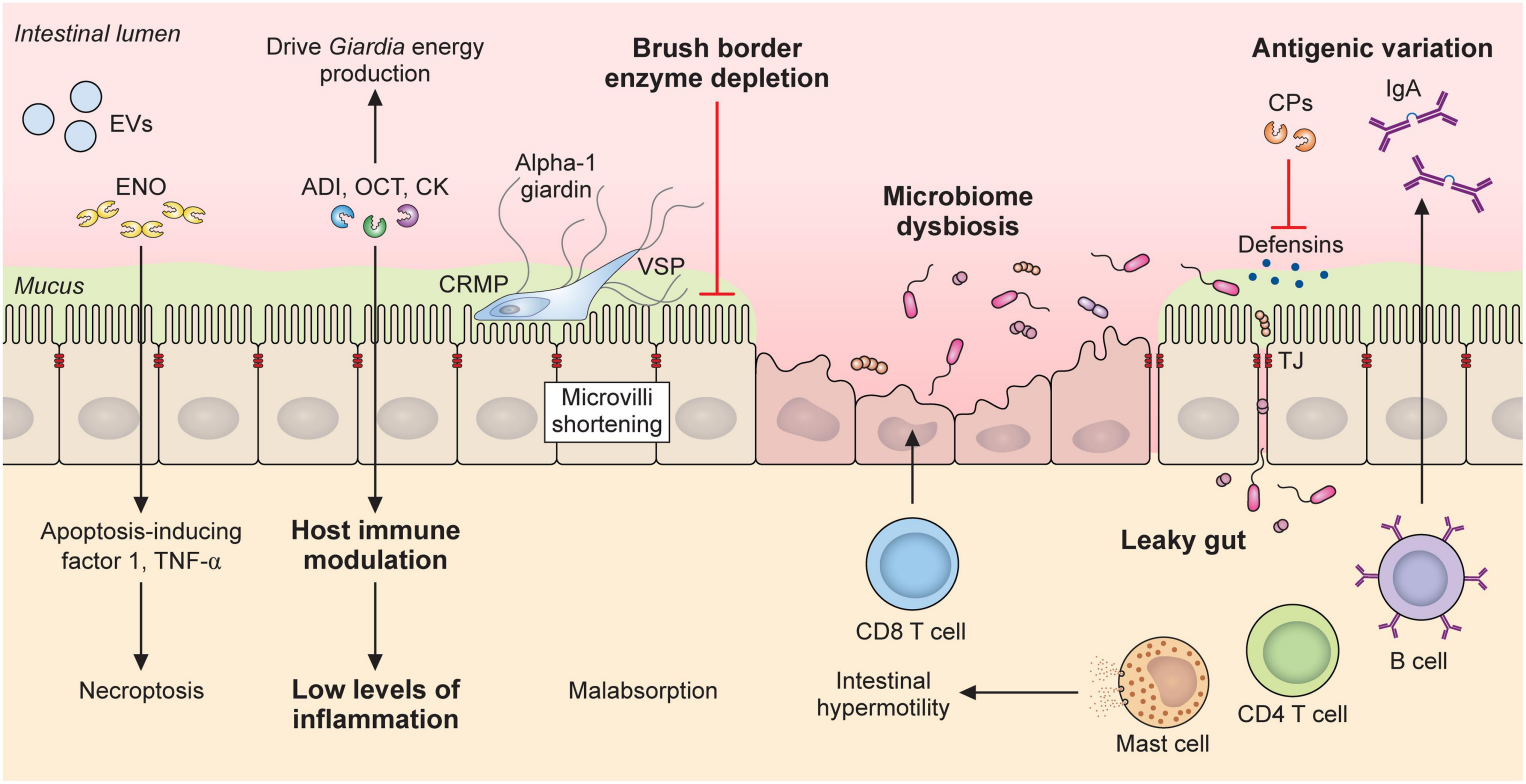
*Giardia*’s interaction with host cells. *Giardia* trophozoites attach quickly to host cells after excystation using surface-associated lectins like alpha-1 giardin and later, when it is fully assembled, the ventral disk. Attachment and secretion, both extracellular vesicles (EVs) and secretory proteins (SPs), disrupt the brush border and shorten the microvilli. Infiltration of CD8 T cells after release of chemokines from the intestinal epithelial cells (IECs) can also affect the brush border. The destruction of the brush border induces malabsorption. Secreted cysteine proteases (CPs) cut and loosen the mucus layer and destroy tight junction proteins, defensins, antibodies (sIGA) and biofilms of intestinal bacteria. Disruption of the tight junctions, together with induction of apoptosis, increases the intestinal permeability, causing a leaky gut problem. The leakage between IECs can introduce bacteria from the microbiome into the submucosa, inducing immune activation and potentially post-infectious problems like IBS. The arginine metabolizing enzymes arginine deiminase (ADI), ornithine carbamoyl transferase (OCT) and carbamate kinase (CK) reduce the local arginine level, which inhibits proliferation of IECs and immune cells. It also reduces production of nitric oxide (NO). Infiltrating mast cells degranulate and induce intestinal hypermotility. Secreted enolase (ENO) can activate plasminogen and induce necroptosis. Antigenic variation of variant-specific surface proteins (VSPs) and cysteine-rich membrane proteins (CRMP) evade the sIgA response, causing chronic infections.

Attachment of *Giardia* trophozoites seems to mainly occur via the ventral disk, but there are also reports of lectin-mediated attachment [58–60]. The annexin-like protein alpha-1 giardin or taglin [60,61] seems to act in parallel with the ventral disc to enhance epithelial adhesion (Figure 2 and Table 1). Its ability to bind intestinal mucins and enterocyte glycans suggests a lectin-like function that complements disc-mediated attachment. This type of non-disk‑mediated binding can be important in the earliest stages of infection since the newly excysted parasite, the excyzoite (Figure 1), lacks a fully assembled ventral disk when it establishes the infection [61]. The earlier mentioned disk-mutants can be valuable in addressing how much of the *Giardia* attachment is lectin-mediated.

### Disruption of intestinal epithelial barrier integrity

Physical interaction between *Giardia* trophozoites and the apical membrane of enterocytes initiates a cascade of brush border damage (Figure 2) driven by mechanical forces, secreted proteins and host immune responses [62]. The integrity of the intestinal epithelium depends on the apical junctional complex (AJC), with tight junctions (TJs), adherens junctions (AJs), and desmosomes (Figure 2) serving as the principal regulators of paracellular permeability. These junctions normally restrict the passage of undigested food particles, bacteria, and microbial products from the intestinal lumen into underlying tissues [63]. However, evidence from human infections, *ex vivo* biopsies, *in vitro* models, and murine studies demonstrates that *Giardia* attaches strongly to the IECs and secretes a diverse array of enzymes and effector molecules that disrupt TJ and AJ proteins, thereby increasing epithelial permeability (Figure 2 and Table 1) and causing a “leaky gut” [64–66].

Mechanistically, *Giardia* disrupts TJ integrity through both contact-dependent and contact-independent mechanisms [30]. Attachment of live trophozoites induces rapid cytoskeletal rearrangement and redistribution of TJ proteins, whereas excretory-secretory products (ESPs) alone can delocalize claudin-1, occludin, and zona occludens-1 (ZO-1) without direct contact. Secreted cysteine proteases directly cleave TJ components (Figure 2) and dismantle the apical junctional complex, an effect that is inhibited by protease inhibitors [67]. In parallel, *Giardia*-induced activation of myosin light chain kinase (MLCK) promotes actomyosin contraction, further destabilizing TJ architecture [68]. This combined disruption of barrier function increases paracellular permeability, enabling leakage of luminal antigens and translocation of commensal bacteria, thereby contributing to inflammation and the pathophysiology of giardiasis [69].

### Apoptosis of enterocytes

Enterocyte apoptosis (Figure 2) is a central and tightly linked mechanism of intestinal barrier disruption in giardiasis, primarily driven by caspase-3 activation, which connects cell death to TJ disorganization and increased permeability. Early studies showed that blocking caspase-3 prevents both apoptosis and TJ damage, establishing a unified pathway from apoptosis to barrier failure [70]. *Giardia* primarily activates the intrinsic (mitochondrial) apoptosis pathway through reactive oxygen species (ROS) generation, which drives mitochondrial dysfunction, shifts the Bax/Bcl‑2 balance, promotes cytochrome c release, and triggers caspase‑9 and caspase‑3 activation. Blocking ROS or preventing uptake of parasite‑derived secreted products markedly reduces this apoptotic response [71]. Additionally, *Giardia* induces ER-stress and activates the unfolded protein response, causing p53-independent cell cycle arrest and reduced epithelial renewal, which compounds tissue damage [72]. The extrinsic pathway also contributes via TNFR1 and caspase-8 activation, while inflammatory cell death mechanisms such as pyroptosis further amplify mucosal stress [71,73]. Collectively, these interconnected pathways drive epithelial loss, impaired regeneration, and sustained barrier dysfunction (Figure 2 and Table 1).

### Brush border damage and microvillus shortening

Infection with *Giardia* trophozoites causes both localized and diffuse changes to the intestinal brush border (Figure 2) through mechanical, immune-mediated and secretory mechanisms. The direct interaction between the contracted ventral disks of trophozoites and the apical layer of enterocytes results in localized microvillus effacement [49]. This is characterized by physical shortening and deformation of the microvilli, which reduces local absorptive capacity. Transmission electron microscopy has demonstrated clear indentation of the enterocyte membrane directly beneath attached trophozoites, indicating a contact-dependent and spatially limited damage to the microvilli [30]. At the same time, a diffuse process driven by the host’s immune response (primarily by CD8(+) T cells) causes widespread microvillus shortening throughout the small intestine [74] and the intestinal microbiome contributes to this CD8(+)T cell response [75]. This microvillus blunting reduces the absorptive surface area and impairs the activity of enzymes (lactase, maltase, sucrase, peptidases and lipase) embedded in the intestinal cell surface that are needed to digest sugars, proteins and other nutrients [76]. These localized changes can also disrupt the organization of lipid rafts and the normal arrangement of brush border enzymes [77].

Giardial excretory-secretory products have also been suggested to affect brush border activities, but the data is limited [78]. Different parasite isolates cause different levels of brush border defects in gerbils, with assemblage B and A1 showing higher pathogenicity than assemblage AII and E isolates [79]. Thus, *Giardia* can cause defects in brush border enzymes in several different ways, but further studies are needed to clarify the molecular mechanisms behind these effects and what roles *Giardia* factors, the immune system and the intestinal microbiome play. The deficiency of brush border disaccharidase can result in osmotic diarrhea and persistent post-infectious lactose intolerance [80,81], suggesting that brush border deficiencies can have effects in acute and post-infectious disease.

### Malabsorption and nutrient deficiencies

The effacement of microvilli, disruption of tight junctions, deficiency in brush border enzymes, luminal competition between trophozoites and host transport processes, and alteration of bile acid availability in giardiasis collectively produce a broad malabsorption syndrome [8,62,82]. Fat malabsorption (steatorrhea) is the dominant clinical feature, resulting from reduced lipase activity, trophozoite interference with micelle uptake, and bile salt depletion, collectively producing calorie deprivation [83]. Malabsorption of fat-soluble vitamins (A, D, E, K) and vitamin B12 and folate produces manifestations ranging from night blindness and coagulopathy to megaloblastic anemia, generally reversible with treatment [11]. These deficits are particularly consequential in children, among whom giardiasis produces dose-dependent linear growth deficits, increased gut permeability, and cognitive impairment, with depletion of key amino acids further contributing to prolonged stunting [84]. In infected children, serum zinc, iron, and selenium levels are reduced, correlating with poor growth [85–87]. Notably, it is also suggested that zinc deficiency caused by giardiasis may impair hippocampal and cerebellar development in early life, providing a potential explanation for associated cognitive delays [88]. There is still limited knowledge about parasite factors that affect nutrient uptake in the host, and further studies are needed since malabsorption in children is a major problem, even during asymptomatic *Giardia* infections [13].

### Giardia and the intestinal microbiome

The interaction between *G. intestinalis* and the host’s gut microbiome plays an important role in the pathophysiology of giardiasis [89]. Notably, preexisting bacterial microbiome diversity determines susceptibility to *Giardia* infection in mice, with less‑diverse microbiomes conferring greater vulnerability [90]. Depletion of intestinal bacteria in experimental models has severe effects on infection dynamics and the host’s immune response [90–92]. However, although numerous studies have reported associations between *Giardia* infection and alterations in gut microbiome composition, results have been inconsistent [12,13,93–99]. Experimental animal infections generally demonstrate pronounced shifts in bacterial communities following infection, whereas human studies have reported effects ranging from substantial microbial alterations to only minor taxonomic changes or no detectable differences after adjustment for age, diet, geography, antibiotic exposure, and co-infections [12,13,93–99]. This is not surprising, considering the many variables in human infections, and most likely the effect of *Giardia* on the microbiome will be context dependent. However, details of G*iardia*’s interaction with the microbiome have been obtained in experimental systems. Probiotic bacteria like *Lactobacillus* spp. can have a protective effect against *Giardia* infections in mice, but this depends on the nutritional status of the animal [100]. *Giardia* modulates bile acid metabolism in young mice, affecting growth [92]. Furthermore, a high-fat diet exacerbates infection severity through preexisting low-grade inflammation and dysbiosis, with fatty acids exerting direct pro-growth effects on trophozoites, underscoring the role of host dietary patterns in modulating disease severity [69]. *Giardia*-induced dysbiotic biofilms sustain epithelial invasion, tight junction disruption, and overproduction of IL-1β independent of the parasite, perpetuating barrier dysfunction beyond parasite clearance [94]. This dysbiosis also disrupts intestinal mucin O-glycosylation in a time-dependent and regiospecific manner, with transplantation of dysbiotic microbiota into germ-free recipients recapitulating these changes, establishing the microbiota as a causal intermediary linking infection to sustained mucosal vulnerability [101]. The gut microbiome protects the host against intestinal infections via colonization resistance, nutrient blocking and immune system priming. This is also true for *Giardia*, but further studies are needed on the role of the whole microbiome, not only bacteria, and their interactions during *Giardia* infections.

### Induction of intestinal hypermotility

Intestinal hypermotility in giardiasis develops in a delayed, adaptive immune-dependent manner, occurring only in immunocompetent hosts as a coordinated protective response [8,102,103]. This response is largely mediated by mucosal mast cells (Figure 2), which release histamine, tryptase, prostaglandin E2, and 5-HT (5-hydroxytryptamine or serotonin) to modulate smooth muscle activity and sensory signaling. Protease-activated receptor-2 activation contributes to visceral hypersensitivity, while neuronal nitric oxide synthase also plays a role in both motility changes and parasite clearance [103–105]. *Giardia* disrupts the enteroendocrine signaling by reducing the 5-HT–producing enterochromaffin cells while increasing cholecystokinin (CCK)-secreting cells. This hormonal shift causes excessive levels of postprandial CCK, leading to dyspeptic symptoms (chronic indigestion and stomach pain) that can persist long after the infection is cleared [106]. In addition, the barrier dysfunction permits bacterial translocation and induces persistent enteric neuronal activation such as histamine-mediated TRPV1 sensitization. This creates a permanent state of long-term gut-hypersensitivity leading to PI-IBS [11,107,108]. These physiological changes lead to lasting clinical consequences, including chronic fatigue and various systemic issues. Studies show that affected individuals often have a significantly higher burden of these conditions and reduced quality of life for years after the initial *Giardia* infection [109–111].

### Clinical manifestations

Although *Giardia* is a noninvasive intestinal parasite, it produces a broad clinical spectrum ranging from asymptomatic carriage to acute or chronic diarrheal illness, with some patients developing post-infectious sequelae, including irritable bowel syndrome, chronic fatigue, and impaired growth [10,112]. A high prevalence of asymptomatic colonization is illustrated by a study of Nigerian school-aged children in which *Giardia* was detected in 77.9% of fecal samples, with 82.6% of infected individuals remaining asymptomatic. This finding supports the view of *Giardia* as a pathobiont capable of occupying a commensal-like niche in the gut [12,113].

The diverse clinical outcomes of *Giardia* infection likely arise from variations in host genetics and immunity, parasite assemblage, and composition of the gut microbiome, making the underlying disease mechanisms complex and not yet fully understood. Although the parasite adheres tightly to the intestinal epithelium and induces changes to the villus microenvironment, mechanical attachment alone does not explain the observed tissue damage. Instead, pathogenesis appears to result from a combination of parasite-derived virulence factors, including secreted factors (Figure 2), the host immune response, and interactions with the gut microbiome [12,105]. Histopathological findings further highlight this complexity, as intestinal biopsies often show villous atrophy and blunting with minimal overt inflammation, and tissue damage may occur at sites distinct from parasite localization [114]. Collectively, these observations indicate that giardiasis is driven by multifactorial and still incompletely understood mechanisms that remain under active investigation. This complexity also requires more complex experimental models that involve parasite, host and microbiome at the same time.

## Virulence determinants in *Giardia*

As *Giardia* trophozoites engage intimately with the host, mainly IECs, they deploy a diverse array of virulence factors, including proteins involved in surface variation and immune evasion, metabolic competition, attachment and motility (Figure 2 and Table 1). In the following sections, we discuss the majority of known or suggested parasite-derived virulence factors involved in the pathogenesis of giardiasis.

### Cysteine-rich surface proteins and antigenic variation

The surface of every *Giardia* trophozoite is covered by a dense layer of surface proteins, mainly cysteine-rich proteins from two major families: variant-specific surface proteins (VSPs) and high-cysteine membrane proteins (HCMPs) or cysteine-rich membrane proteins (CRMP) [115]. The two families share broad structural features rooted in cysteine richness and membrane topology, yet they differ substantially in their structural organization and phylogenetic classification. CRMPs include at least 94 members, with about a dozen being actively secreted during host-cell contact [116], while VSPs form a larger family of 136 members, though only one variant is typically expressed on the surface at a time in any given parasite population or culture (Figure 2). In addition to these two major groups, *Giardia* also encodes other cysteine-rich proteins, including high-cysteine proteins (HCPs) and tenascin-like proteins (TLPs), now included in secretory cysteine-rich proteins, SCRPs, with 16 members [9,115,117]

#### Variant-specific surface proteins (VSPs)

VSPs are integral membrane proteins with highly variable extracellular domains (Table 1) [115]. They contain multiple CxxC motifs and a zinc‑finger-like motif, along with hydrophobic regions and palmitoylated C-terminal segments that help anchor them in the membrane [1,118]. Additionally, they include a conserved transmembrane domain (TMD) and a short cytoplasmic tail (CT) of only five amino acids (CRGKA). VSPs have N-terminal signal sequences and are transported via the ER to the parasite surface. The presence of multiple protein-disulfide isomerases in the ER promotes disulfide formation and compact folding of the VSPs [115]. VSPs coat the entire surface of *Giardia* trophozoites and represent the dominant antigens recognized by the host’s immune system [115].

*Giardia* controls its VSPs through a precisely regulated system that allows only one major VSP to be present on the parasite surface at any given time. This selective expression is not completely understood, but it has been suggested to involve RNA interference (RNAi)-like mechanisms, including RNA-dependent RNA polymerase (RdRP), Dicer, and Argonaute, which silence non-expressed VSP genes via siRNAs or miRNAs (such as miR2) [115,119,120]. There is also involvement of chromatin structure in the regulation of VSP expression [121,122]. It is possible that the small RNA and chromatin structure regulation are connected, but more studies, preferentially using knock-out mutants of key regulatory proteins and single-cell analyses, are needed to reveal the regulatory mechanism.

Although the molecular signals and mechanisms that initiate VSP switching in trophozoites have remained a long-standing puzzle, this process is essential for immune evasion, allowing the parasite to alter its surface antigens and escape host immune recognition. This is also a major reason for *Giardia*’s tendency to give cyclic symptoms and chronic infections. During interactions with IECs, trophozoites exhibit strain-specific preferred VSP expression patterns, including VSP123 (WB), VSP8 (P-1), VSP160 (NF), and VSP-H7 (GS/M), highlighting the dynamic and adaptive nature of antigenic variation [123]. While earlier models suggested that antigen switching occurred passively or through antibody-mediated killing [124], recent findings reveal a more dynamic process: antibodies binding to VSPs do not kill the parasite but instead induce clustering of these proteins into specialized lipid membrane microdomains [125]. This clustering creates mechanical stress that triggers the release of microvesicles containing the original VSP, effectively removing the immune target from the cell surface. At the same time, this process initiates a calcium-dependent switch (a mechanism termed -*Antigen Removal Coupled to Switching*- or ARCS) to a different VSP variant, allowing the parasite to rapidly change its antigenic identity. Additionally, the released extracellular vesicles may act as decoys, diverting the host immune response while the parasite completes antigenic switching [125]. Altogether, this integrated system seems to link RNAi-based gene regulation, chromatin structure, membrane dynamics, and immune interactions into an efficient adaptive strategy that enables *Giardia* to evade immune detection and maintain persistent infection.

VSPs are not only immune targets; they also influence how the host responds to infection. One major role of the VSPs is to protect against proteolytic activities in the upper small intestine [126]. Certain VSPs, such as VSP1267, VSP9B10, and VSPH7, have been suggested to activate host innate responses via toll-like receptor-2 (TLR2) and TLR4-dependent mechanisms after purification as recombinant proteins from insect cells [126,127]. If mixtures of purified VSPs are given to gerbils in an oral formulation, cytokine responses show a biphasic profile in the intestine [128]: an early induction of Th1 (γ‑interferon, interleukin‑1β, IL-6 and tumor necrosis factor), Th17 (IL-17) and Th2 (IL-4) cytokines, together with intestinal alterations typical of inflammation, followed by a shift toward a predominant Th2 (IL-5) response, likely associated with a counter‑regulatory mechanism. Immunization with an oral vaccine comprising the entire repertoire of VSPs after Dicer knock-down specifically showed high levels of IL-17, IL-6, IL-4 and IL-5, without obvious signs of inflammation [128]. Immunized animals developed local intestinal secretory IgA (S-IgA) and systemic serum IgG humoral immune responses, showing that VSPs can be used in oral vaccination [128]. VSPs in the VSP9B10 group have received attention due to their functional and pathogenic roles in *Giardia* [129]. Members such as VSP9B10A have been suggested to contribute to parasite adhesion, as antibodies against it reduce trophozoite attachment [30,130,131], while VSP9B10B appears to be involved in encystation [132]. VSP9B10A (GL50803_101074) has been identified in the *Giardia* secretome and exhibits proteolytic activity like cathepsin B, enabling it to damage epithelial cells and disrupt cellular integrity, establishing it as a conditional virulence factor [131,133]. This cytotoxic effect is directly mediated by the protein and can be neutralized by specific antibodies [131]. Together, these findings highlight that, beyond immune evasion, certain VSPs may function as active virulence factors, though targeted inhibitors remain largely undeveloped [123,134–136].

Nevertheless, certain VSPs, such as VSPAS7, have been shown to attenuate inflammatory responses. Recently, Sun et al. reported that VSPAS7 overexpression in mouse peritoneal macrophages suppresses *Giardia*-induced NF-κB signaling, as indicated by reduced phosphorylation of p65 and IκBα, along with decreased ERK/MAPK pathway activation, ultimately lowering the secretion of IL-6, IL-12p40, and TNF-α [127]. This functional duality might enable *Giardia* to modulate host immune responses, promoting immune evasion while preventing excessive inflammation that could be detrimental to parasite survival.

#### Cysteine-rich membrane proteins (CRMPs)

The CRMPs or HCMPs (Table 1) form a structurally diverse family of cysteine-rich proteins and, unlike VSPs, multiple CRMPs are simultaneously surface-expressed by individual trophozoites, and approximately a dozen are actively secreted during trophozoite-epithelial cell interactions [115,116]. They have N-terminal signal sequences, are transported via the ER, and contain multiple CxxC and CxC motifs [115,116]. Among the most differentially expressed genes during host‑cell contact, members of the CRMP family are prominently up-regulated, with their expression further modulated by histone acetylation and free iron availability in the cells [116]. This suggests intestinal iron levels regulate the deployment of multiple putative virulence factors in *Giardia* [116]. Transcriptomic analyses of four *Giardia* strains co-cultured with intestinal epithelial cells showed that up to 17 different CRMPs are upregulated upon host contact [116]. However, only a small subset is secreted into the host environment, with three CRMPs detected in the WB strain and two in the GS strain. Notably, even when the same strains and cell lines were used, the specific CRMPs identified in the secretome varied between studies, underscoring that the progression from gene expression to protein secretion is tightly controlled and strongly context-dependent [137,138]. This variation reflects the inherent flexibility in how CRMPs are deployed across different isolates and in response to environmental conditions [139]. Structural damage to intestinal epithelial cells is caused not only by trophozoite attachment but also by the release of excretory-secretory products, although the specific roles of individual CRMPs in epithelial injury are not yet fully understood. The presence of both shared and genotype-specific CRMPs across assemblages A, B, and E, as well as within sub-assemblages of known genomic background [134,135], strongly suggests that this protein family plays an important role in host adaptation and contributes to the variability in disease outcomes seen in human *Giardia* infections.

#### Secretory cysteine-rich proteins (SCRPs)

The SCRPs contain a group of tenascin-like proteins which are a newly recognized class of secreted virulence factors of *Giardia* implicated in the disruption of intestinal tissue repair (Table 1). Approximately 20 SCRP-encoding genes are distributed across *Giardia* strains WB, GS, and P15, and they share EGF-like domains with VSPs and HCMPs [115]. Multiple proteomic studies confirm SCRP secretion across both human-infective assemblages, with up to seven distinct SCRPs identified in the WB secretome, establishing them among the most abundant secreted proteins during host–parasite interaction [137,138]. The proposed pathogenic mechanism is EGF-receptor activation and IEC apoptosis induction [140]. Despite their consistent presence in the trophozoite secretome, the precise roles of giardial SCRPs in pathogenesis remain unresolved, and experimental studies using purified native and recombinant proteins are needed to clarify their contribution to disease.

### Excretory-secretory products (ESPs)

Trophozoites release ESPs, and this contributes to epithelial damage and disease development (Figure 2). The ESPs contain free proteins, secretory proteins (SP), and extracellular vesicles (EVs) [137,141]. Proteomic studies indicate that, during interactions with IECs, trophozoites release more than 200 proteins via active secretion, extracellular vesicles, surface shedding, and most likely also via cellular leakage [137,138]. Many of the proteins in the *Giardia* secretome lack classical secretory signal sequences, and this was initially used as an argument for leakage-induced release. However, unconventional protein secretion (UPS) has been found in all types of eukaryotes. It usually does not require signal sequences and bypasses secretory organelles such as the ER and Golgi [142]. Several secretory pathways are active in *Giardia* simultaneously [143]. The parasite lacks a true Golgi-apparatus and uses a UPS pathway involving peripheral vacuoles (PVs) that briefly fuse with the plasma membrane to exchange extracellular materials [144]. Thus, UPS can be an important mechanism for the release of ESPs. The ESPs include metabolic enzymes, surface-associated molecules, cytoskeleton proteins and soluble mediators. They interact with cellular receptors and extracellular matrix components and induce apoptosis, metabolic competition with host cells, cytopathic damage, and disruption of intestinal homeostasis [30,144–146]. EV release in *Giardia* has been suggested to be an important virulence mechanism in *Giardia* (Figure 2 and Table 1), enabling delivery of multiple virulence factors to host cells. Two major EV populations are described: small vesicles (SVs, exosome-like particles; <100 nm) and large vesicles (LVs, microvesicle-like particles; 100–400 nm), each carrying diverse proteins with potential roles in virulence. These EVs incorporate multiple virulence-associated cytosolic and membrane-associated proteins, including several alpha-giardins, VSPs, other high cysteine-rich proteins and metabolic enzymes [145,147]. The formation of EVs in *Giardia* is controlled by peptidyl-arginine deiminases (PADs) and inhibiting PAD activity blocks large-EV production and sharply reduces trophozoite adhesion, indicating EV production as a plausible target for anti-virulence drugs [148]. Vesicles can also deliver small RNAs that alter gene expression in bacteria [149], parasites [150,151] and host cells [141] in the nearby environment.

#### Secreted proteases

*Giardia* secretes a diverse set of extracellular proteases that serve as key virulence factors, disrupting the intestinal epithelium and thereby promoting parasite attachment and invasion, aiding nutrient uptake, and modulating host immune responses [30]. Cysteine proteases (CPs), particularly the cathepsin B-like (*cat*B) superfamily, are among the best-characterized secreted virulence determinants of *Giardia* trophozoites (Figure 2 and Table 1) and have been identified as central mediators of multiple pathogenic processes [136]. The genome of *G. intestinalis* (reference clone WBC6) encodes up to 25 cysteine proteases, predominantly of the cathepsin type, including eight cathepsin-L, nine cathepsin-B enzymes with endopeptidase activity, three cathepsin-B enzymes with an occluding loop associated with dipeptidyl activity, and a cathepsin-C-like enzyme known as the encystation-specific cysteine protease (ESCP) [9,152]. Giardial CPs are synthesized as inactive precursors and become active after removal of the N-terminal pro-domain. They contain a catalytic site with the conserved *Cys-His-Asn* triad and an S2-binding pocket. Their mechanism involves a deprotonated cysteine attacking the carbonyl carbon of the substrate, forming a thioester intermediate that is hydrolyzed to yield a carboxylic acid group. As typical cysteine proteases, their activity produces cumulative and coordinated effects on host proteins when expressed or secreted by trophozoites. Notably, these proteases are differentially expressed during trophozoite growth and encystation, with cathepsin B-like proteases being the most abundantly expressed [153,154]. Overall, the *Giardia* cathepsin family appears to function in both specialized and overlapping roles across distinct biological processes and cellular compartments [9].

Cysteine proteases exhibit strong proteolytic activity and play central roles in the pathogenesis of *Giardia*. The protease secretion increases markedly in the presence of live epithelial cells, indicating that host contact enhances cysteine‑protease release [137,138,155]. During interaction with IECs, these enzymes disrupt tight junctions, induce enterocyte apoptosis, degrade host immune components including chemokines and immunoglobulins, and contribute to mucus depletion and gut microbiota dysbiosis. The three principal secreted CPs (CP14019, CP16160, and CP16779) disrupt apical junctional complexes by altering the localization and integrity of TJ- and AJ proteins, including claudins, occludin, JAM-1, β-catenin, and E-cadherin, thereby weakening epithelial barrier function and increasing paracellular permeability [67]. Among them, giardipain-1, also called CP2 or CP14019, is the most thoroughly characterized CP. The precursor of giardipain-1 (a 33-kDa multi-domain protein) is activated by removing a 76-amino-acid pro-region, resulting in a 25-kDa mature enzyme with potent gelatinolytic and pro-apoptotic activity. Purified mature giardipain‑1 induces membrane blebbing and apoptosis in IEC‑6 and MDCK monolayers, highlighting its strong cytotoxic action. Once released in its active form from trophozoites, giardipain-1 localizes to the intestinal brush border and reproduces the junctional disruption and accelerated enterocyte apoptosis characteristic of natural giardiasis [156].

*Giardia* CPs also cleave the microvillus-associated cytoskeletal protein called villin through a host myosin light chain kinase (MLCK)-dependent pathway, directly linking protease activity to brush border disruption and epithelial cytoskeletal remodeling [68]. Moreover, CP-mediated alterations in intestinal mucin glycosylation, with reductions in sulfomucin production, render the mucus barrier progressively more susceptible to protease-mediated breakdown, representing a novel self-amplifying mechanism of *Giardia* pathogenesis [101]. In parallel, *Giardia* CPs also act as potent immunosuppressive and microbiome-disrupting effectors (Figure 2). They cleave key immune substrates such as immunoglobulins and defensins (Figure 2), thereby weakening both innate and adaptive immune responses and promoting parasite persistence. At the epithelial surface, CPs suppress innate immune signaling by degrading key chemokines including CXCL1-3, CXCL8, CCL2, and CCL20. Although CXCL8 is initially upregulated during *Giardia* infection, its rapid proteolytic degradation is suggested to dampen neutrophil and mononuclear cell recruitment and to contribute to immune evasion. The combined activities of CPs in degrading junctional proteins and chemokines can partly explain the low levels of inflammation seen during giardiasis, while simultaneously creating the conditions for diarrhea and post-infectious problems such as irritable bowel syndrome and food allergies. Nonetheless, CPs also degrade key mucosal defense molecules, including secretory IgA and β-defensin 1, the latter specifically cleaved by CPs 14,019, 16,160 and 16,779, enabling trophozoite survival in the small intestinal lumen despite active innate immune responses [67,157]. Alongside this, *Giardia* CPs contribute to gut microbial dysbiosis. *Giardia* trophozoites or their ESPs alter human colonic biofilms, reduce their thickness, and shift bacterial composition toward a Clostridiales-dominated microbiome. It has been observed that, when transferred to germ-free mice, these dysbiotic biofilms trigger epithelial ZO-1 disruption, apoptosis, pro-inflammatory cytokine induction, and bacterial translocation [9]. In addition, *Giardia* CPs attenuate granulocyte chemotaxis in the intestinal lumen, significantly reducing pro-inflammatory cytokine responses to *C. difficile* toxins, *Salmonella* sp., and *Escherichia coli* [136]. These effects are diminished by the CP inhibitor E-64, implicating *Giardia* CPs in degrading biofilm extracellular-matrix components and facilitating infection [94,158]. The roles of the different CPs in *Giardia* are clearly overlapping, and the CPs are involved in many different processes, making interpretation of data difficult. CRISPR mutagenesis of selected CPs, combined with *in vitro* and *in vivo* host‑cell interactions, can clarify specific roles. It will also be important to study the CP repertoires and expression patterns in different *Giardia* assemblages since initial genomic analyses have already identified assemblage-specific differences [136]. Finally, the broad role of the giardial CPs makes them interesting targets for chemotherapy.

#### Metabolic enzymes as moonlighting virulence factors

*Giardia* secretes intracellular metabolic enzymes that have been suggested to acquire new extracellular roles once released into the host environment. These “moonlighting” enzymes, originally involved in glycolysis or arginine metabolism, have the potential to function as virulence effectors during the host–parasite interaction, which suggests that *Giardia* can repurpose core metabolic proteins to enhance infection [159,160]. The release of these metabolic enzymes is not constitutive but is specifically upregulated by contact with intestinal epithelial cells. Using established co-culture models, intestinal ligated loops, and a proteomics approach, three *Giardia* proteins, namely, arginine deiminase (ADI), ornithine carbamoyl transferase (OCT), and enolase (ENO), previously recognized as immunodominant antigens during acute giardiasis (Figure 2), were initially identified in the *Giardia* secretome [30]. Recent, deeper analyses of the *Giardia* secretome show that these three enzymes are mainly released as free, secreted proteins in the SP fraction and not in extracellular vesicles (EVs) [141]. The enzymes localize to the trophozoite cytoplasm and the inner plasma-membrane surface and have been suggested to interact with components of the UPS machinery [144]. Their expression peaks at 1.5 hours and then declines, a biphasic pattern indicative of a regulated, host-induced secretory response [123,161].

##### Giardia enolase (gENO)

*Giardia* enolase (gENO; GL50803_11118) is the most abundant glycolytic enzyme detected in trophozoite secretomes (Figure 2) during interaction with Caco-2 and HT-29 IECs, as well as in murine intestinal loops infected with GS strain *in vivo* [162]. Within trophozoites, gENO shows a punctate cytoplasmic pattern, but during host‑cell co‑incubation it redistributes toward the anterior pole and is actively secreted into the extracellular space. Enolase is the predominant enzyme released during interaction with Caco-2 cells and in murine intestinal loops among the five glycolytic enzymes detected in the *Giardia* secretome (fructose bis-phosphate aldolase (gFBPA), glyceraldehyde-3-phosphate dehydrogenase (gG3PDH), phosphoglycerate kinase (gPGK), phosphoglucomutase (gPGM), and enolase (gENO)) [9].

Bioinformatic and experimental analyses suggest that dimeric gENO binds human plasminogen (HsPLG), a fibrinolytic system component, through lysine residues (Lys66, Lys186, Lys188) that engage the Kringle K4 and K5 domains of HsPLG. Subsequent evidence showed that endogenous gENO is actually secreted as a monomer and can activate HsPLG to generate plasmin activity, a serine protease that promotes extracellular matrix degradation [9]. A recombinant form of gENO induces cytopathic damage in IEC-6 monolayers, including increased cell separation, vacuolization, and a caspase-3-independent death pathway involving apoptosis-inducing factor 1 and TNF-α expression [163]. Because TNF-α participates in signaling pathways leading to programmed necrosis (necroptosis), gENO may contribute to early stages of chronic intestinal pathology, which features epithelial barrier loss and microbiota dysbiosis commonly associated with post-giardiasis complications [69]. The double‑stranded RNA virus Giardiavirus (GLV) can potentially modulate the pathological damage and immune response in mice, and gENO is associated with GLV replication [164]. Knowledge of the putative role of GLV during human *Giardia* infections and its spread among *Giardia* isolates is currently limited. Thus, further studies of the role of GLV in *Giardia* biology and disease-induction are needed.

##### Giardia arginine deiminase (gADI)

The *Giardia* arginine deiminase (gADI; GL50803_112103) is the most abundantly secreted protein during trophozoite–IEC interaction and represents one of the most multifunctional metabolic virulence factors (Figure 2). Intracellularly, gADI operates within the arginine dihydrolase pathway (ADHP), converting L-arginine to L-citrulline and generating ATP for trophozoite survival under anaerobic conditions [20]. Arginine metabolism is also important in cyst-formation [20] and gADI changes intracellular localization during the encystation process [165]. Once secreted, however, gADI depletes extracellular L-arginine, the sole substrate for host nitric oxide synthase, in a strategy termed “warginine.” This cytoplasmic enzyme is released in large quantities upon contact with IECs and is directly responsible for arginine depletion. Loss of extracellular arginine alters host immunity by increasing TNF-α and decreasing IL-10 production in dendritic cells, and by suppressing T-cell proliferation. Thus, beyond its metabolic role in trophozoite energy production, gADI functions as a bona fide virulence factor that modulates host immune responses [20,166].

In vitro experiments with recombinant ADI show that the secreted enzyme suppresses innate defenses by lowering nitric oxide production in cytokine-stimulated Caco-2 cells, as indicated by reduced nitrite levels in the culture medium. This NO inhibition weakens neutrophil killing, reduces smooth muscle contractility (potentially aiding trophozoite retention in the proximal small intestine), disrupts encystation and excystation signaling, and dampens mucosal inflammation, creating a permissive niche for persistent colonization [20,21,167]. In addition, secreted gADI directly modulates immune cell function; monocyte-derived human dendritic cells stimulated with LPS in the presence of catalytically active gADI exhibit elevated TNF-α alongside reduced IL-10, IL-12p40, CD83, and CD86, as well as inhibition of mTOR-S6-kinase signaling, a profile consistent with immunosuppressive dendritic cell polarization [166]. In addition, gADI activates rat mast cells to release IL-6 and TNF-α, potentially contributing to epithelial damage and chronic inflammatory sequelae of giardiasis [168]. Recent data suggest that genetically encoded differences in ADI from different *Giardia* assemblages affect the activity of the enzyme [20]. Genetic knockout- mutants have been generated for ADI [20] and this can potentially be used to connect ADI and arginine metabolism to assemblage-associated differences in growth, differentiation and pathogenicity.

##### Giardia ornithine carbamoyltransferase (gOCT)

Ornithine carbamoyl transferase (gOCT; GL50803_10311) is consistently detected as a major secreted protein during trophozoite–epithelial interactions (Figure 2) in both assemblage A (WB) and assemblage B (GS) strains [137,162]. Within trophozoites, gOCT catalyzes the second step of the ADHP, generating L-ornithine that fuels the surface arginine/ornithine antiporter and sustains gADI-driven competitive arginine depletion. It also produces carbamoyl-phosphate, which is used in ATP production by the third enzyme in the ADHP pathway, carbamate kinase (CK). The CK enzyme (Figure 2) is also often found in *Giardia* secretomes [137] but it has so far not been studied as a virulence factor. Although the extracellular role of secreted gOCT remains unresolved, its strong serological recognition in serum and milk from infected individuals across endemic and non-endemic regions highlights substantial exposure during natural infection and supports its potential as a diagnostic or vaccine candidate [169,170]. The crystal structure (PDB 3GRF) shares only 30% sequence identity with the human enzyme, indicating potential for the development of species-selective inhibitors [171].

Altogether, these secreted metabolic enzymes form a combined set of virulence factors that exploit the dependence of *Giardia* on host arginine and glucose. They drive energy production, suppress host immunity, and damage the intestinal epithelium. Their consistent detection across various strains and their strong immunogenicity in infected humans could make them attractive targets as biomarkers for diagnostic tools and for anti-giardial drug and vaccine development. The recent availability of genetic knock-out mutants of certain enzymes can be combined with animal experiments to make even stronger connections between *Giardia*’s metabolic enzymes and pathogenicity.

### Cytoskeletal proteins

*Giardia* relies on a highly specialized cytoskeletal apparatus to establish infection in the small intestine, where sustained attachment to the epithelium is required for colonization, persistence, and pathogenesis [28]. It also needs to swim and reattach in an environment where up to 9 L of liquid passes daily. This process is coordinated by a complex network of structural and regulatory proteins that mediate the assembly and functional integrity of the ventral disk (Table 1), median body, and flagellar apparatus (Table 1), which collectively regulate motility, adhesion, and cellular morphogenesis [28]. In addition to their canonical structural functions, several of these parasite-derived cytoskeletal proteins also appear in the secretome during *Giardia*-host interactions, suggesting additional functions in host communication and virulence modulation. The giardins, a specialized family of disk-associated cytoskeletal proteins (DAP), are key contributors to the virulence machinery of the parasite [30,172].

Giardins are a family of low-molecular-weight cytoskeletal proteins (≈29–38 kDa) that localize to the ventral disk, the primary attachment organelle of *Giardia*. They include several subtypes, such as alpha, beta, gamma, and delta-giardins (Table 1), which stabilize the spiral array of microtubules, microribbons, and crossbridges that define the disc’s cup-shaped architecture. In addition to structural integrity, giardins are essential for parasite virulence; disrupting these proteins compromises epithelial attachment and directly impairs intestinal colonization. They also contribute to motility, cytokinesis, and developmental transitions through cytoskeletal remodeling [30,173].

Among the giardins, alpha-giardins are the best-characterized subgroup and show annexin-like, Ca++ dependent phospholipid binding that anchors the ventral disc cytoskeleton to the plasma membrane. This positioning maintains a tight seal with the host epithelium while allowing for flexible attachment and detachment. A prominent example, alpha-1 giardin, binds acidic phospholipids and glycosaminoglycans [61] and is released extracellularly despite lacking classical secretion signals (Figure 2 and Table 1). Its enrichment in a UPS complex associated with PVs suggests that alpha-1 giardin acts both as a cargo and a mediator in a non-canonical *Giardia* secretion pathway [144,174,175]. The presence of alpha-giardins in secreted fractions, lipid-raft-like domains, and extracellular vesicles suggests roles beyond structural support, potentially contributing to host-cell modulation or signaling. Consequently, several giardins are highly immunoreactive during acute infection [169]. Both alpha-1 and beta-giardin are recognized by human antibodies across assemblages A and B, highlighting their exposure to the host immune system and their potential as diagnostic markers or vaccine candidates [176]. Together, these features position giardins as key elements of *Giardia*’s virulence architecture, where annexin-related proteins have been adapted to support adhesion, flagellar motility, structural resilience, secretion and host interaction. A deeper understanding of giardin biology, both as DAPs and as part of the different flagella, is therefore likely to yield important insights into both the cell biology and pathogenesis of this early-diverging eukaryotic parasite.

### Other toxin‑like molecules

*Giardia* trophozoites also release toxin‑like factors that induce key features of giardiasis, including intestinal hyperperistalsis, fluid secretion, and epithelial injury. Two early‑identified candidates of such molecules are CRP136 (VSP188) and gESP58, both detected in excretory–secretory products from cultured trophozoites [9]. CRP136/VSP188, a variant surface protein encoded by GL50803_137613, contains sarafotoxin‑like repeats homologous to endothelin precursors and is predicted to activate endothelin receptor A (ETAR), thereby enhancing intestinal smooth‑muscle contraction and motility. gESP58, a 58-kDa glycoprotein from the Portland-1 strain (assemblage A), exhibits broad enterotoxin-like activity. It increases epithelial permeability in skin assays, induces fluid accumulation in rabbit ileal loops and mouse sealed gut, causes cytopathic effects in HEp-2 monolayers, and agglutinates erythrocytes [177–179]. Patient sera recognize gESP58, and the protein binds a putative 41-kDa receptor on mouse enterocytes; this interaction is blocked by ganglioside M1 (GM1) receptor, consistent with a cholera toxin-like pathway involving cAMP elevation, PKA activation, and chloride efflux. However, the protein has never been identified, preventing further characterization. This suggests that, although *Giardia* lacks classical toxin genes, CRP136/VSP188 and gESP58 can elicit effects like those of enterotoxins. Although their detection is not consistent in recent secretome datasets, these molecules are interesting virulence candidates with potential structural and therapeutic relevance [9,180].

## Host immune response against *Giardia*

The immune response to *Giardia* is a complex interplay between parasite virulence, the intestinal environment, and innate and adaptive mechanisms [181,182]. Innate immunity forms the first barrier against *Giardia* trophozoites, yet the parasite rapidly undermines these defenses through targeted virulence mechanisms. Trophozoites attach to the epithelial surface and release an array of ESPs that degrade mucus, defensins, and tight-junction proteins, weakening barrier integrity and enabling bacterial translocation [68] (Figure 2). Cysteine proteases such as giardipain-1 degrade intestinal mucin MUC2 and antimicrobial peptides/defensins [157], while ESPs disrupt claudins, occludins, and E-cadherin, increasing permeability and causing cytoskeletal damage. In response, intestinal epithelial cells produce chemokines including CXCL1-3, CXCL8, CCL2, and CCL20, though *Giardia* proteases can degrade these chemokines and limit neutrophil recruitment [67] (Figure 2). In addition, metabolic enzymes such as ADI and OCT further suppress nitric oxide production and impair T-cell proliferation, linking metabolic competition to innate and adaptive immune dysregulation [20,137,156]. Reactive oxygen species (ROS) are induced and released by the IECs, and *Giardia* upregulates systems to deal with the ROS [183]. Secreted EVs have been shown to induce the NLRP3 inflammasome [184] and the inflammasome is also activated by recombinant alpha-giardins [185] and *Giardia* MIF [186].

Adaptive immunity in mice relies on a coordinated response from intestinal IgA and CD4^+^ T-cell, with VSP-specific secretory IgA and IL-17A-producing Th17 cells playing central roles in parasite control [181,182,187–189]. IgA targets semiconserved regions of VSPs and drives antigenic variation [190,191], while antibody binding can trigger antigen removal and switching [125]. IL-17A is essential for epithelial defense and IgA production, and IL-17RA-deficient mice exhibit impaired induction of antimicrobial genes (eg. mannose‑binding protein 2, dual oxidase 2, gasdermin C and C2, and polymeric immunoglobulin receptor) and prolonged infection [76,187,188]. *Giardia* counters these responses through protease-mediated degradation of IgA and IgG and by skewing immune cells (dendritic cells, macrophages, and T cells) toward IL-10 production, suppressing Th1/Th17 polarization [113,192–194]. This interplay between protective IgA/Th17 activity and regulatory IL-10 signaling determines whether infection is cleared efficiently or progresses to chronic or recurrent disease [1,181,189]. *Giardia* induces a type 2 mucosal immune response in mice, with antigen-specific Th2 cells, increased expression of type 2 cytokines (IL-4,IL-13) with infiltration of IL-10‑producing Th2 and Treg cells, but also goblet‑cell hyperplasia and increased IL-17A and IL-25 expression [113]. This facilitates chronicity and also confers a STAT6-dependent protection from inflammatory pathologies [113], showing an important role of intestinal parasitic infections like *Giardia* in the balance of mucosal immune responses.

Host susceptibility and disease outcome are mostly dependent on nutritional status, age, microbiome composition, immune competence, and co-infections [195]. Children in endemic regions experience frequent re-infections and gradually acquire partial immunity, whereas susceptible adults are more prone to symptomatic disease and post-infectious sequelae [111,196]. The microbiota strongly influences pathology: germ-free animals develop infection without disease, and *Giardia*-induced dysbiosis promotes inflammation and may contribute to post-infectious IBS [12,197]. Co-infections can either enhance antimicrobial defenses through NLRP3 activation or exacerbate malnutrition and inflammation [198,199]. Trophozoite metabolic fitness at the time of infection has recently been identified as a key determinant of disease outcome, which dictates whether host immune signaling is activated or suppressed [200]. This shows the complexity of *Giardia*’s interaction with the host’s immune system. Further experiments *in vivo* using mutants defective in specific immune‑evasion pathways are needed to show connections with specific immune responses in the host.

## Summary and future outlook

Despite major progress in understanding *Giardia* biology, several questions remain unanswered. Progress has been made in understanding pathogenicity, and certain virulence factors have been described, but the connections between specific disease-causing mechanisms and virulence factors in *Giardia* are still few and poorly defined. The *Giardia* secretome has been suggested to be an important factor in pathogenesis but it is still not known what it affects and how. Proteomic studies list more than 200 secreted proteins, but fewer than one‑fifth have been functionally validated, and there is little overlap between different studies, making it difficult to determine what the most important activities are. Many predicted cytotoxic or immunomodulatory cysteine-rich proteins and released metabolic enzymes remain to be studied *in vivo*. The mechanisms that drive chronic infection and post-infectious complications also remain unclear. There is limited understanding of persistent colonization based on *Giardia* assemblage type, VSP regulation, or protease profiles, highlighting the need for longitudinal studies that integrate immune profiling, VSP tracking, and microbiome analysis. Parts of the mechanism of VSP switching are known, but much is still missing. The discovery of antibody-driven VSP switching raises new questions about immune memory and cross-assemblage protection, which are central to vaccine design. Isolates from different assemblages and sub-assemblages need to be studied in more detail; most of the data have been generated using one isolate. Host genetic susceptibility is another unexplored area, particularly variation in innate immune receptors, IgA pathways, and tight‑junction genes. Rising nitroimidazole treatment failures further highlight the need for genomic studies of drug-tolerant isolates and for rigorous evaluation of combination therapies and identification of new drug candidates.

The recent technological developments and increasing availability of molecular and cellular tools and new model systems now make it possible to gain deep knowledge about *Giardia*’s pathogenicity and virulence. Clearly, *Giardia* research is entering an exceptionally dynamic and exciting phase.

## Data Availability

Data sharing is not applicable to this article since no data were generated or analyzed in this study.
